# Improved inpatient deterioration detection in general wards by using time-series vital signs

**DOI:** 10.1038/s41598-022-16195-2

**Published:** 2022-07-13

**Authors:** Chang-Fu Su, Shu-I Chiu, Jyh-Shing Roger Jang, Feipei Lai

**Affiliations:** 1 Graduate Institute of Biomedical Electronics and Bioinformatics, National Taiwan University, Taipei, Taiwan, ROC; 2 Division of Medical Quality, En-Chu-Kong Hospital, New Taipei, Taiwan, ROC; 3 Department of Anesthesia, En-Chu-Kong Hospital, New Taipei, Taiwan, ROC; 4 Department of Electronic Engineering, Asia Eastern University of Science and Technology, New Taipei, Taiwan, ROC; 5 Department of Computer Science, National Chengchi University, Taipei, Taiwan, ROC; 6 Department of Computer Science and Information Engineering, National Taiwan University, Taipei, Taiwan, ROC; 7 Department of Electrical Engineering, National Taiwan University, Taipei, Taiwan, ROC

**Keywords:** Health care, Risk factors

## Abstract

Although in-hospital cardiac arrest is uncommon, it has a high mortality rate. Risk identification of at-risk patients is critical for post-cardiac arrest survival rates. Early warning scoring systems are generally used to identify hospitalized patients at risk of deterioration. However, these systems often require clinical data that are not always regularly measured. We developed a more accurate, machine learning-based model to predict clinical deterioration. The time series early warning score (TEWS) used only heart rate, systolic blood pressure, and respiratory data, which are regularly measured in general wards. We tested the performance of the TEWS in two tasks performed with data from the electronic medical records of 16,865 adult admissions and compared the results with those of other classifications. The TEWS detected more deteriorations with the same level of specificity as the different algorithms did when inputting vital signs data from 48 h before an event. Our framework improved in-hospital cardiac arrest prediction and demonstrated that previously obtained vital signs data can be used to identify at-risk patients in real-time. This model may be an alternative method for detecting patient deterioration.

## Introduction

Although uncommon, in-hospital cardiac arrest (IHCA) has a high mortality rate. In the United States, approximately 200,000 cardiac arrests are treated annually in hospitalized patients, and the event rate of cardiac arrest is about 0.92 per 1000 bed days^1^. The post-discharge survival rate for patients with cardiac arrest is nearly 25% in the United States and less than 20% worldwide^2, 3^. Intra-arrest factors, including whether the arrest was witnessed or monitored and whether it occurred during daytime hours, were reported to be associated with increased odds of survival^4^. Identifying at-risk patients has become critical because post-cardiac arrest survival rates are associated with care team awareness.

Various early warning scoring systems have been used to detect hospitalized patients at high risk of deterioration^5–11^. Such scoring systems are generally developed by selecting appropriate variables associated with prediction outcomes. Most early warning scoring systems, such as the Modified Early Warning Score (MEWS)^12^, use vital signs, including temperature, heart rate, respiratory rate, and blood pressure, for clinical judgments. Because the areas under the receiver operating characteristic curve (AUROC) for MEWS have been below 0.7 in many studies, research has been conducted to include more types of clinical data, including laboratory results, demographics, and heart rate variability, to increase prediction performance^5–7, 13–15^. These methods have been demonstrated to be more accurate, resulting in fewer false alarms and more favorable detection. However, the methods may not be feasible in care units where these clinical data are not regularly measured. Information about the research to predict IHCA is presented in Table 1.

**Table 1:** Comparison of research for IHCA detection in hospitals.

	Churpek et al.^5^	Green et al.^6^	Bartkowiak et al.^7^	Kwon et al.^8^	Kim et al.^9^	Cho et al.^10^
Year of publication	2016	2018	2018	2018	2019	2020
Care unit	Ward	Ward	Ward (surgical)	Ward	ICU	ward
Vital sign data	8 h (single)	4 h (single)	(single)	8 h (multiple)	6 h (multiple)	8 h (multiple)
AUROC of MEWS	0.698	0.698	0.750	0.603	0.746	0.684
AUROC of best model	0.801 (Random Forest)	0.801 (eCART)	0.790 (eCART)	0.850 (DEWS)	0.896 (FAST-PACE)	0.865 (DEWS)
SBP	X	X	X	X	X	X
HR	X	X	X	X	X	X
RR	X	X	X	X	X	X
BT	X	X	X	X	X	X
DBP	X	X	X		X	
SpO2	X	X	X		X	
AVPU score	X	X	X			
Other clinical data	22	21	26	0	3	0
Total variables	29	28	33	4	9	4

The increasing application of artificial intelligence and machine learning (ML) systems has fundamentally altered the biomedical field from molecule to disease level^16^. ML facilitates the automatic analysis of highly complex data and produces meaningful results. ML models can improve prediction accuracy with the same data or reduce features with the same performance^17^. Cho and Kwon used vital signs over the past 8 h to develop a deep learning-based early warning score to predict deterioration in patients in general wards accurately. Some research used ML with continuous vital signs to predict deterioration in intensive care units (ICUs)^9^. However, continuous vital signs measurements may not be available in general wards.

Therefore, in this study, we sought to develop a more accurate machine-learning model (the time series early warning score [TEWS]) for predicting clinical deterioration using only heart rate, systolic blood pressure, and respiratory data. These vital signs which are regularly measured in general wards. This model may be an alternative to the MEWS system.

## Methods

### Ethics declarations

This retrospective cohort study was approved by the Institutional Review Board (IRB) of the En-Chu-Kong Hospital (IRB number: ECKIRB1071001). We confirm that all experiments were performed in accordance with relevant guidelines and regulations. The data retrieved from electronic health records (EHRs) were de-identified by an IT specialist and could not be linked to the patients’ identity by the research team. The need for written informed consent was waived and confirmed by the En-Chu-Kong Hospital IRB (ECKIRB1071001) because this was a retrospective cohort study with de-identified data.

### Study setting and population

The study population comprised inpatients from a community general hospital. The study data set was EHRs of the adult inpatients (aged ≥ 20 years) who visited the hospital between August 2016 and September 2019. Each patient’s information was anonymized and de-identified before analysis.

#### Data sources

We used five vital signs as predictor features: systolic blood pressure (SBP), diastolic blood pressure (DBP), heart rate (HR), respiratory rate (RR), and body temperature (BT). Medical staff measured these vital signs regularly, at least two to three times per day during the day, night, and early morning. We defined the time window (TW) measurement as 8 h. Therefore, 1 day comprised three TWs. We considered features measured during each TW; each TW had five vital signs measurements.

The hospital data were divided according to date into a derivation (August 2016–November 2018) and a validation set (December 2018–September 2019). The derivation and validation sets were used to develop the TEWS and to determine the TEWS parameters, respectively. We used AUROC and area under the precision-recall curve (AUPRC) values for binary classification. The characteristics of the study population are listed in Table 2.

**Table 2:** Characteristics of the study population.

Characteristic	Model derivation	Model validation
Study period	2016/8–2018/11	2018/12–2019/9
Total patients	11,762	5103
Patients with IHCA	81	37
Age, mean ± SD	63.8 ± 19.9	63.7 ± 20.5
Male sex (%)	5875 (49.9)	2293 (44.9)
Weight, mean ± SD	63.2 ± 14.7	63.3 ± 17.6
Respiratory rate (1TW), mean ± SD	18.9 ± 4.1	19.1 ± 5.0
Diastolic blood pressure (1TW), mean ± SD	73.6 ± 15.2	72.3 ± 20.1
Systolic blood pressure (1TW), mean ± SD	133.2 ± 31.0	135.0 ± 40.8
Temperature (1TW), mean ± SD	36.7 ± 4.4	37.5 ± 6.4
Heart rate (1TW), mean ± SD	83.4 ± 21.5	84.9 ± 23.5

#### Outcomes

The primary outcome of interest was cardiac arrest, defined as a loss of pulse with attempted resuscitation. We examined the collected EHRs to identify the exact time of each outcome. We categorized the selected inpatients into positive and negative groups. The positive group contained inpatients with a cardiac arrest event in the general wards. For patients with several cardiac arrest events during their stay at the hospital, we used only the first event. The negative group contained inpatients who did not stay in the ICU and had no cardiac arrest event during the study period.

The TEWS was compared with the MEWS and other classifiers. We then performed a time analysis of the vital signs and predicted whether a patient would be IHCA-positive by using the features recorded in one, three, or six TWs (i.e., 8, 24, or 48 h, respectively).

### Model development

#### Data preprocessing

Because the collected EHRs may have contained human or system errors, our data had the potential to have missing values. For example, medical staff may have failed to measure vital signs during some TWs, leading to missing data in the TWs. To compensate for the missing values, we applied the multiple imputation by chained equations approach^18^. The advantage of this approach is that it not only restores the natural variability of missing values but also incorporates the uncertainty resulting from the missing data, thus enabling a valid statistical inference. In the event of duplicate data for the same TW, we used the maximum value.

Values of the features in our data were distributed over a wide range, which increased the difficulty of training the classifier. Therefore, we used standard scores (commonly referred to as z-scores) to adjust the values of all features.

#### Handing imbalanced data

In many real-life problems, especially in the medical field, data sets are imbalanced; that is, the class distribution in such data sets is severely skewed. Similarly, our data set was imbalanced. However, most machine-learning algorithms are most effective when the number of samples in each class is nearly balanced. Failure to manage imbalanced data sets can adversely affect the performance of classifiers^19^; in machine-learning classifiers, biases in training data sets can lead to minority classes being ignored entirely. Accordingly, imbalanced data sets can be managed through under-sampling (in which samples are deleted from the majority class) and oversampling (in which samples from the minority class are duplicated). In our previous study, we under-sampled IHCA-negative samples for detection. The results indicated that our approach effectively solved the imbalance in the data set used for detecting cardiac arrest^20^.

Accordingly, in the present study, we used a modified weight balancing method in place of an oversampling or under-sampling approach to balance our data set. We used this method when the number of samples in one of our classes was substantially higher than in the other. This method modified the class weights according to the ratio of IHCA-positive to IHCA-negative samples to ensure that all classes contributed equally to the loss. Furthermore, we applied focal loss to balance the weight of our training samples^21^. When an imbalanced data set is used for classification, the majority class is adequately represented in the classification because more data are available for this class; however, the minority class is not sufficiently represented. We applied “focal loss” to prevent this situation. Focal loss assigns relatively high weight to the minority class during training to ensure that the class is adequately represented in the classification. Therefore, we applied focal loss and the weight-balancing method to our imbalanced data in developing the TEWS.

**Figure 1:**
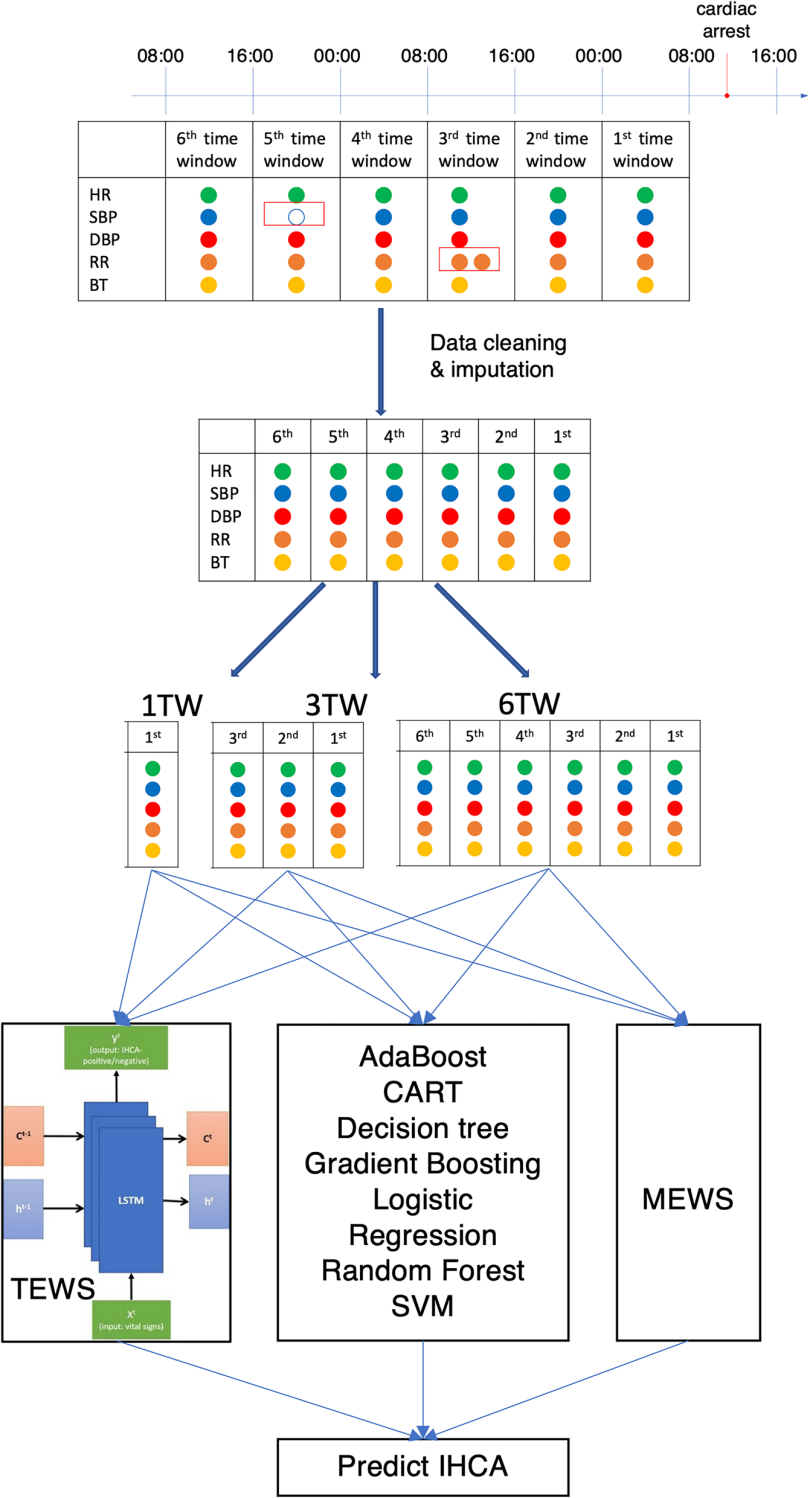
Study workflow. *TW* time window.

We used features obtained in 1, 3, and 6 TWs (i.e., 8, 24, and 48 h, respectively). Each TW contained one set of features. The study workflow is illustrated in Fig. 1.

#### Time series early warning score [TEWS] model

**Figure 2:**
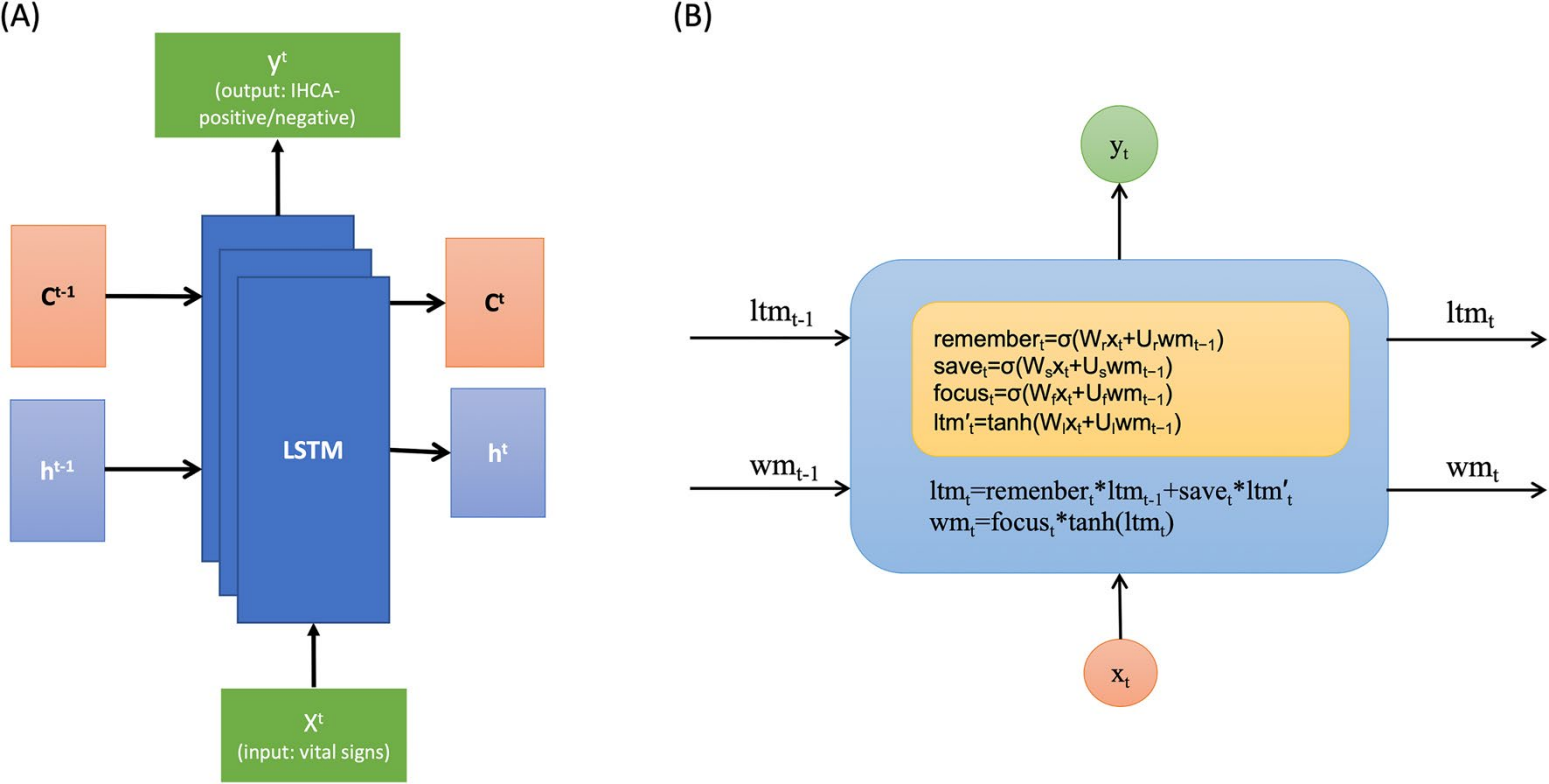
(**A**) TEWS architecture. (**B**) LSTM architecture.

The proposed TEWS comprises three recurrent neural network (RNN) layers with LSTM^22, 23^. An RNN is a neural network with feedback loops, which enable it to process sequential data, such as EHRs^24^. The architecture of TEWS and LSTM is illustrated in Fig. 2. The LSTM unit comprises a cell, an input gate, an output gate, and a forget gate. The cell remembers values over arbitrary time intervals, and three gates regulate the flow of information into and out of the cell. LSTM deals with the time series data well. Therefore, TEWS can adequately process time-series data.

**Figure 3:**
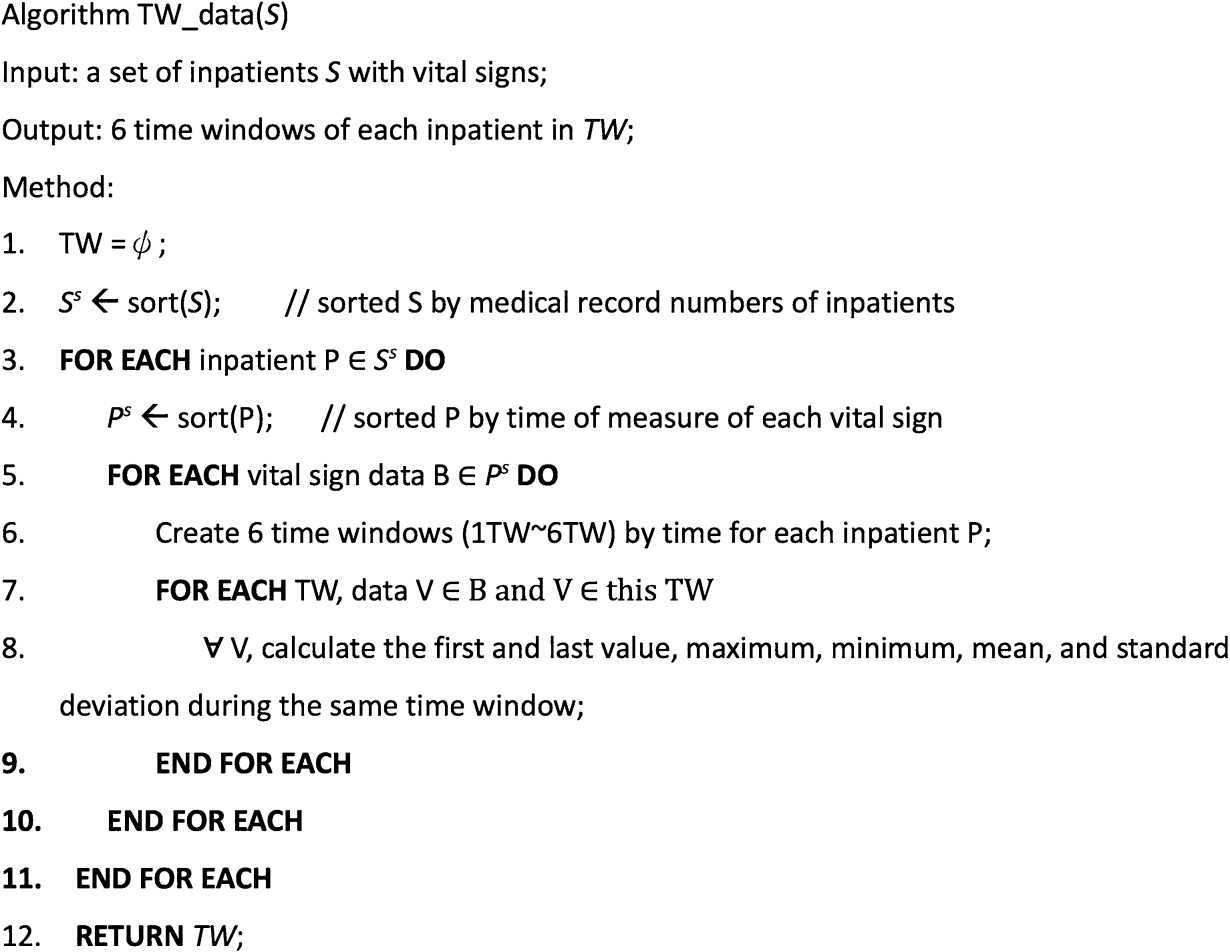
The algorithm for creating time windows.

We train the TEWS model using the training data set and assess TEWS model performance using the validation data set. In our TEWS model, the training and validation data set are split into an 8:2 ratio. Figure 3 presents the algorithm to create six time windows for each vital sign of all inpatients.

### Performance evaluation

#### Benchmarking with contemporary algorithms

We implemented our LSTM-based system by using the scikit-learn package in Python^25^; the neural networks were implemented in Keras, with TensorFlow serving as the backend engine. The scikit-learn package was also used to implement some classification for comparison^26^, namely naïve Bayes^27^, support vector machine (SVM)^28, 29^, AdaBoost^30, 31^, *k*-nearest neighbor^32, 33^, classification and regression tree^34^, and C4.5 decision tree^35^. We also used gradient boosting^36^, logistic regression^37^, and random forest^38^ algorithms. Gradient boosting produces a prediction model in the form of an ensemble of weak prediction models, and it can be considered an optimization algorithm on a suitable cost function^39^. Logistic regression is a statistical model used to model the probability of a specific class. It uses a logistic function to model a binary dependent variable in its basic form. Random forest is an ensemble learning method for classification, regression, and other tasks; it involves constructing a multitude of decision trees during training and outputting a class (that is, the mode of the classes or the mean prediction of the individual trees). Because of our imbalanced data, our proposed TEWS sets class weights according to the ratio of IHCA-positive to IHCA-negative samples. We compared the prediction performance of these classifications and TEWS.

Predicted probabilities were calculated for each observation of validation data set from each derived model to understand the accuracy of results within the context of the literature. The result of MEWS was also calculated. The AUROCs and the AUPRCs were determined according to whether an event occurred within eight hours of each observation because these are standard early warning score comparisons metrics.

### Feature selection

Feature selection involves selecting the best features from a set of valuable features for discriminating between classes. Feature selection can be completed through an elimination process. Feature elimination methods can be broadly classified into filter and wrapper methods. In wrapper methods, the feature selection criterion is the predictor’s performance (i.e., the predictor is wrapped on a search algorithm that will identify the subset with the highest predictor performance). Sequential backward selection (SBS) algorithms are straightforward and greedy search algorithms. An SBS algorithm can be used for feature selection. The algorithm removes one feature from a complete set of features at a time, leading to a minimal decrease in predictor performance. SBS performs most favorably when the optimal subset has fewer features^40, 41^.

## Results

**Figure 4:**
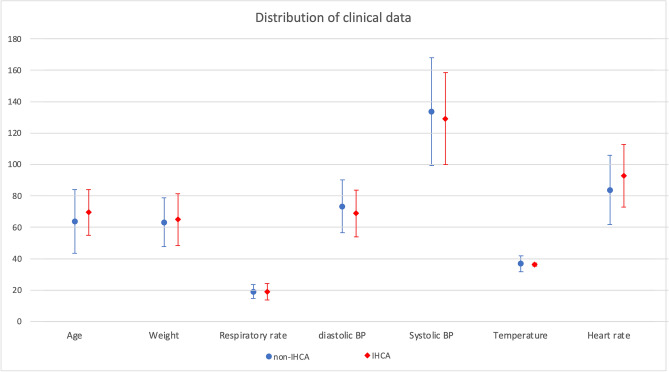
Data distribution of patient vital signs in general ward patients. Mean ± SD. *SD* standard deviation, *IHCA* IHCA-positive group, *non-IHCA* IHCA-negative group.

A total of 16,865 adult admissions were included in this study. 118 (0.7%) of these patients experienced cardiac arrest in a general ward (Table 2).We further describe the characteristics of IHCA-positive and IHCA-negative data in Fig. 4.

We used two tasks to test the performance of our proposed TEWS. We then compared the results of TEWS and these classifications. The tasks are detailed as follows.

### First task

Prediction of IHCA Using Features (Vital Signs) Recorded in One Time Window (8 h), Three TWs (24 h), and Six TWs (48 h).

**Figure 5:**
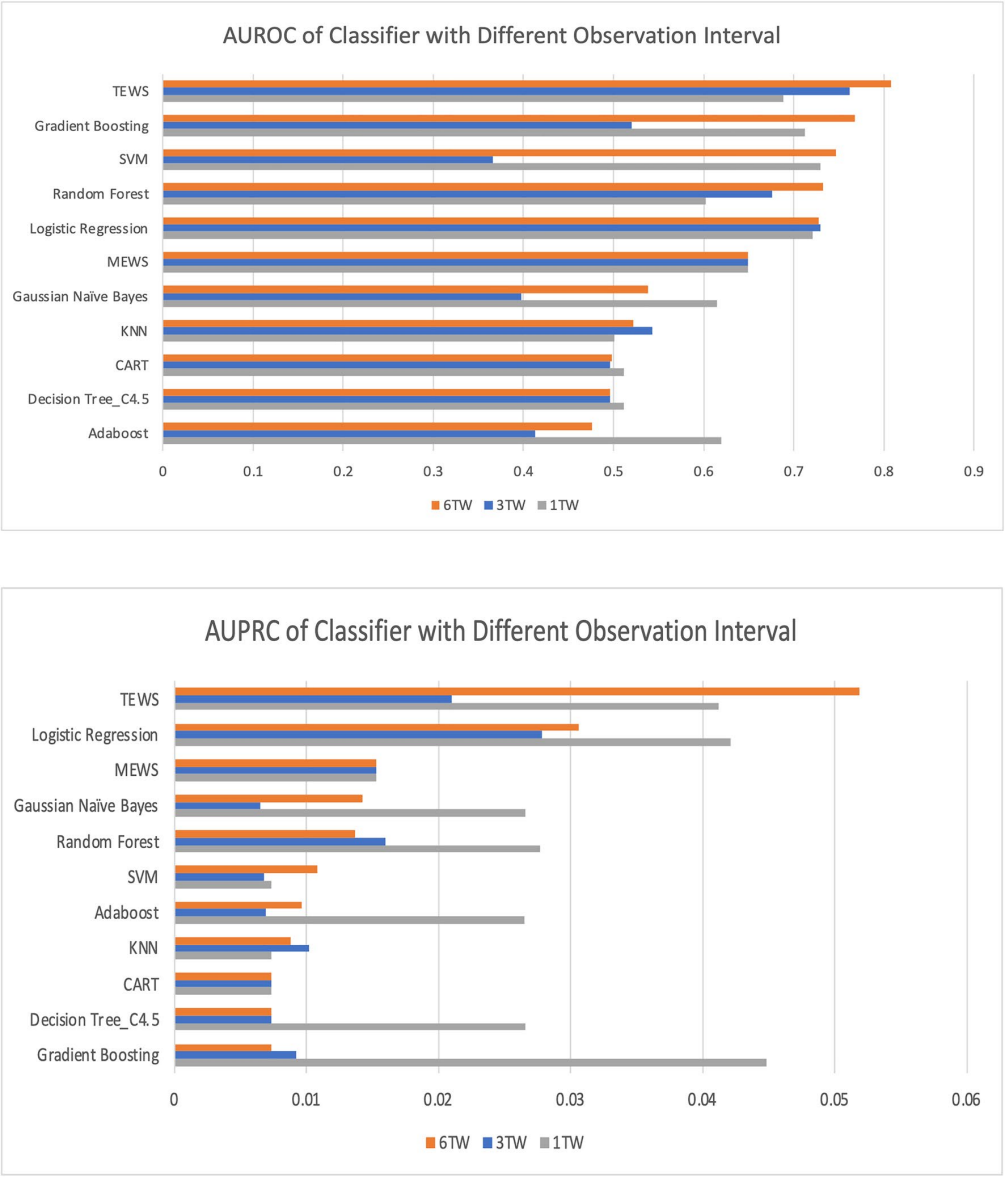
AUROC and AUPRC values for classifiers with one, three, and six time windows, *TW* time window, *TEWS* time series early warning score.

In the 1TW (8 h) group, we applied one set of five vital signs (i.e., features obtained in one TW) to predict IHCA events using the proposed TEWS. The performance of the TEWS model was then compared with that of the MEWS and other classifiers, as displayed in Fig. 5. ROC and PR curve are illustrated in supplementary files. The support vector machine (SVM) and logistic regression algorithms had the highest AUROC values (0.729 and 0.721, respectively), followed by gradient boosting (0.712) and the TEWS (0.688). However, no classifier adequately outperformed the MEWS.

In the 3TW (24 h) group, we applied features recorded in three TWs (24 h) to predict IHCA events using the TEWS. Each TW included a single set of vital signs; therefore, three TWs with five vital signs' measurements contained 15 features. The AUROC value of the TEWS (0.762) was superior to those of the logistic regression (0.730), random forest (0.676), MEWS (0.649), and other algorithms.

In the 6TW group (48 h), we applied features recorded in six TWs (48 h) to predict IHCA events using the TEWS. The AUROC value of the TEWS (0.808) was superior to those of gradient boosting (0.768), SVM (0.747), random forest (0.733), and other algorithms. TEWS performed well regardless of the 1TW, 3TW, and 6TW groups.

Most classification algorithms exhibited similar performance levels when we used features from a single TW. The AUROCs of these models were within 0.62–0.73 (AUROC of MEWS: 0.65). When we used more TWs, some classifications exhibited improved performance levels. Our TEWS demonstrated more favorable prediction in the 6TW (AUROC = 0.808, area under the precision-recall curve [AUPRC] = 0.052) than the MEWS did (AUROC = 0.649, AUPRC = 0.015).

### Second task

Prediction of IHCA Using Features Selected Through Sequential Backward Selection (SBS) algorithm.

**Figure 6:**
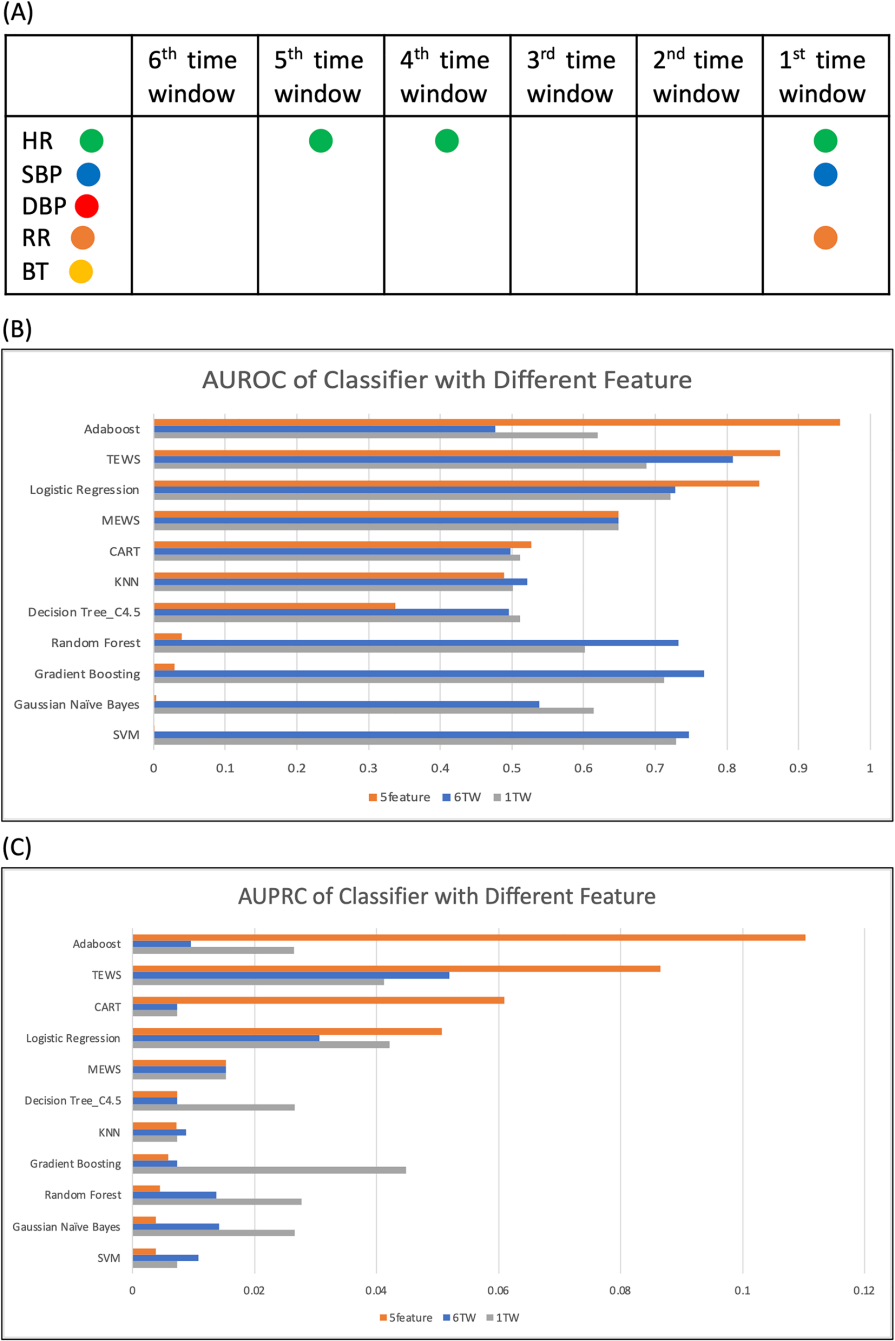
(**A**) Features selected through SBS algorithm. (**B,C**) AUROC and AUPRC values of the classifier with five selected features at one and six TWs; *TW* time window, *TEWS* time series early warning score, *5feature* five selected features.

The TEWS had the most favorable performance in the first task when six TWs (48 h) were included. Because six TWs comprise 30 features, we sought a means of reducing the required features without compromising performance. We selected the most relevant features in the six TWs by using an SBS algorithm. These selected features are presented in Fig. 6. The first TW was the time window closest to the cardiopulmonary resuscitation time for patients who were IHCA-positive. Heart rate, respiratory rate, and systolic blood pressure were the most relevant features for predicting IHCA events. The top five features were heart rate in the first, fourth, and fifth TWs, respiratory rate, and systolic blood pressure in the first TW.

Furthermore, we applied the five selected features to the TEWS model and the other algorithms. The performance of the algorithms using the five features was then compared with that of the MEWS and other classifiers, as displayed in Fig. 6. The TEWS (AUROC = 0.875, AUPRC = 0.087), Adaboost (AUROC = 0.958, AUPRC = 0.110), and logistic regression (AUROC = 0.845, AUPRC = 0.050) achieved their highest performance.

## Discussion

In this study, we used only vital signs in two days to predict cardiac arrest. Our results revealed that the TEWS model using features from six TWs outperformed the other classification algorithms. When the TEWS was implemented using features from six TWs, its prediction performance (AUROC = 0.808, AUPRC = 0.052) was higher than that when it was implemented using features from a single TW (AUROC = 0.688, AUPRC = 0.041) and that of the MEWS (AUROC = 0.649, AUPRC = 0.015). The improved model performance suggests that more information on vital signs data could be obtained from different TWs.

Similar studies have also reported that the essential predictor variables for clinical deterioration are respiratory rate, heart rate, age, and systolic blood pressure^5^. Our study proposed TEWS with only five features from six TWs (respiratory rate, systolic blood pressure in the most recent TW, and three heart rate values in different TWs; AUROC = 0.875, AUPRC = 0.087) outperformed the other classifications. The result indicates that trends in heart rate variation rather than absolute heart rate value alone are essential data.

Our study has several strengths compared with others. First, although some deep learning-based early warning systems can accurately predict patients’ deterioration in intensive care settings, our TEWS can be implemented in general wards or long-term care units. Second, we used a longer observation time (48 h) for vital signs and deep learning-based method to increase the accuracy of predicting cardiac arrest without additional variables. Third, we developed our model using only vital signs. This model can be widely implemented in any system that is equipped for MEWS. The minimum requirement for TEWS is a single personal computer with the capacity for manual entry of vital signs or automatic extraction from EHRs.

Our study has several limitations. First, this was a single-center study at a community general hospital. Therefore, the results may not be generalizable to other settings. Second, our TEWS had the best performance when vital signs from 48 h were included; it did not have a higher prediction performance on the first day of admission compared with other early warning systems. However, prehospital heart rate collected through wearable devices may be an alternative data source for the model. Third, we could not accurately predict several cases of cardiac arrest in our data set. Some cases involved sudden collapses, such as a pulmonary embolism after cesarean section or postoperative airway obstruction with hematoma, which were not predicted. In addition, TEWS could not detect deterioration between two time windows. This is a limitation of noncontinuous vital signs-based prediction models.

## Conclusion

We developed a LSTM-based model using vital signs data from 48 h to predict IHCA. TEWS detected more deteriorations with the same level of specificity as the other algorithms. Our results demonstrate that the 6TW-TEWS and five feature–TEWS more favorably predicted deterioration than the different algorithms did with 1TW (Supplementary Information).

Our framework improved IHCA prediction and demonstrated the feasibility of using only previously obtained vital signs data to detect critical illness in ward patients in real-time. Our TEWS model may be an alternative method for detecting patient deterioration**.**

## Supplementary Information

Supplementary Information.

## Data Availability

The dataset analyzed in this study is available from the corresponding authors on reasonable request and upon approval by the Institutional Review Board (IRB) of the first authors’ institution to share the data. We will share our dataset including vital signs data with 6 time windows. The hyperlink of our training dataset is https://www.cs.nccu.edu.tw/~sichiu/allz_train_6tw.csv. The hyperlink of our test dataset is https://www.cs.nccu.edu.tw/~sichiu/allz_test_6tw.csv.
