# Supplementary material for: Improved inpatient deterioration detection in general wards by using time-series vital signs
Source: Sci Rep. 2022 Jul 13;12:11901. doi: 10.1038/s41598-022-16195-2 (PMC9279370; doi:10.1038/s41598-022-16195-2)

**Supplementary Information**

1. The Receiver Operating Curve of Classifier with 1, 3, 6 Time windows and 5 features.

(TEWS=Time series Early Warning Score.)

1. The ROC curve of 1 Time window


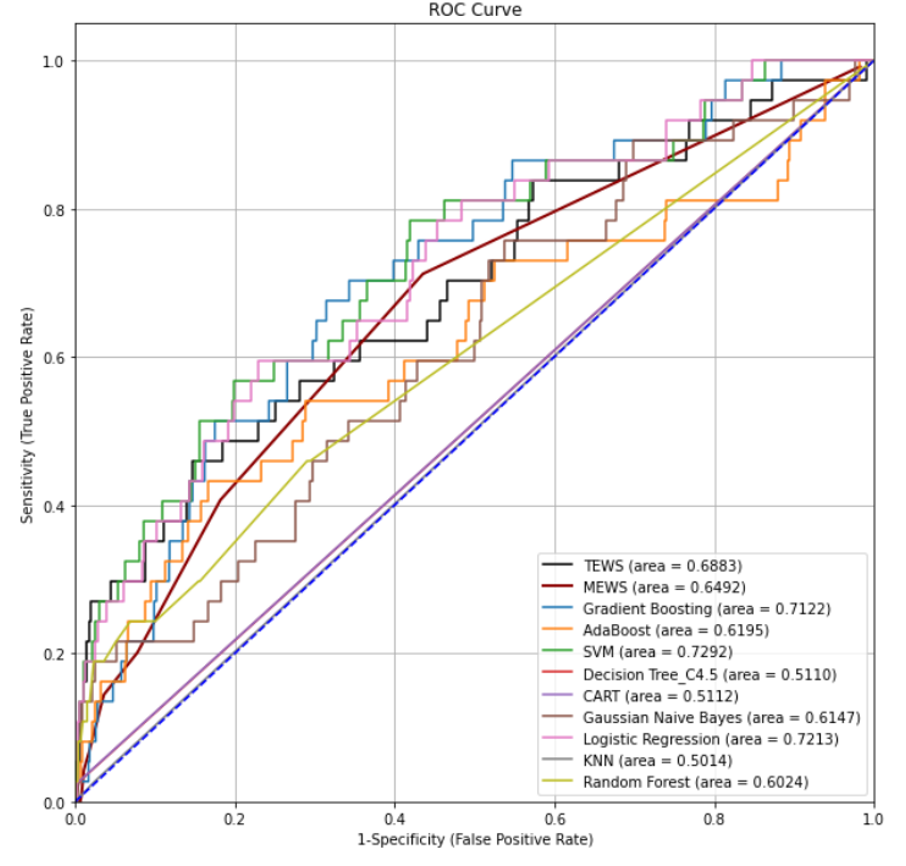


1. The ROC curve of 3 Time windows


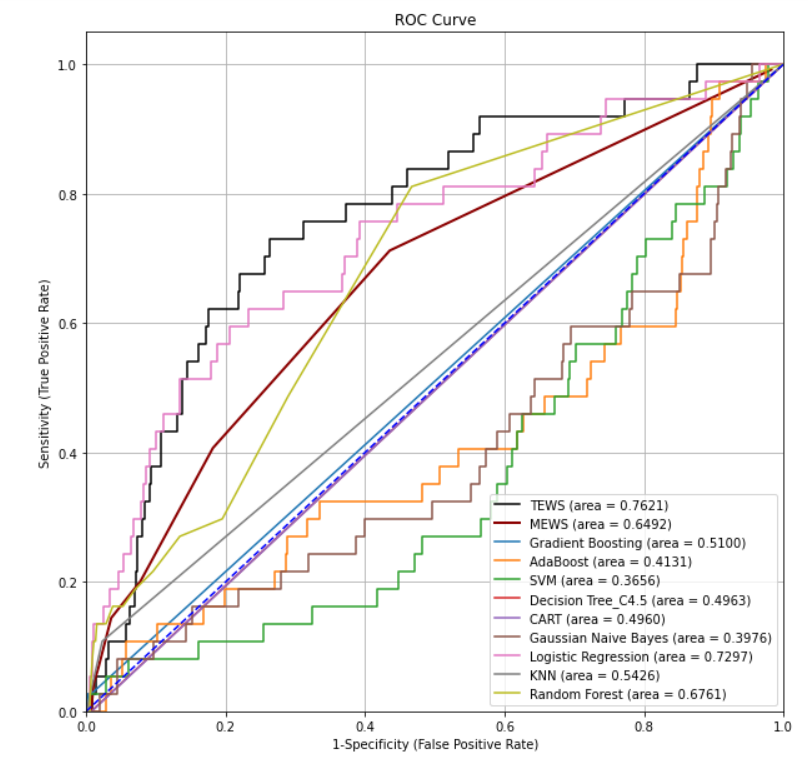


1. The ROC curve of 6 Time windows with 30 features


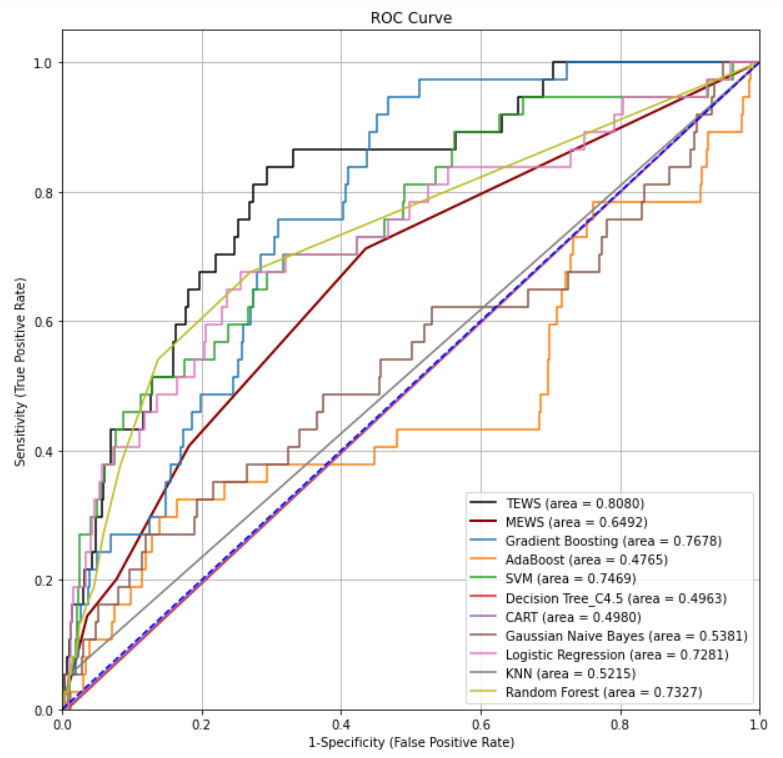


1. The ROC curve of 6 Time windows with 5 selected features
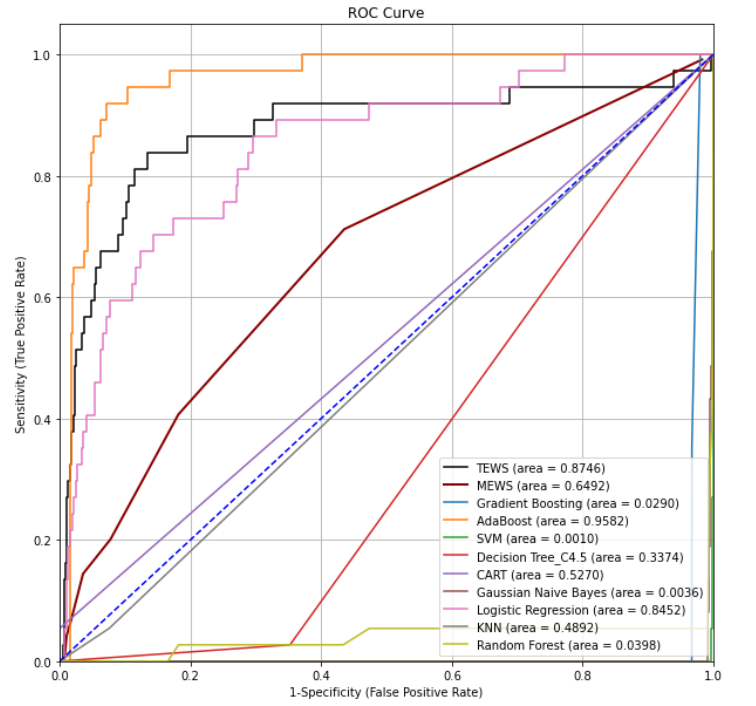

2. The Precision-Recall Curve of Classifier with 1, 3, 6 Time windows and 5 features.

(TEWS=Time series Early Warning Score.)

1. The PR curve of 1 Time window
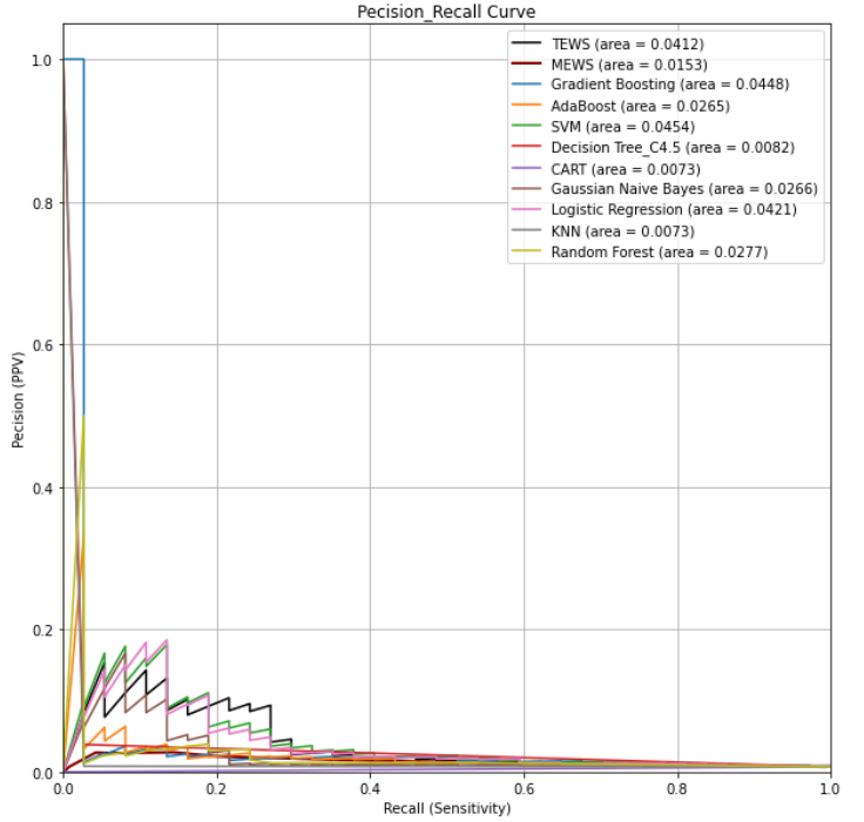

2. The PR curve of 3 Time windows


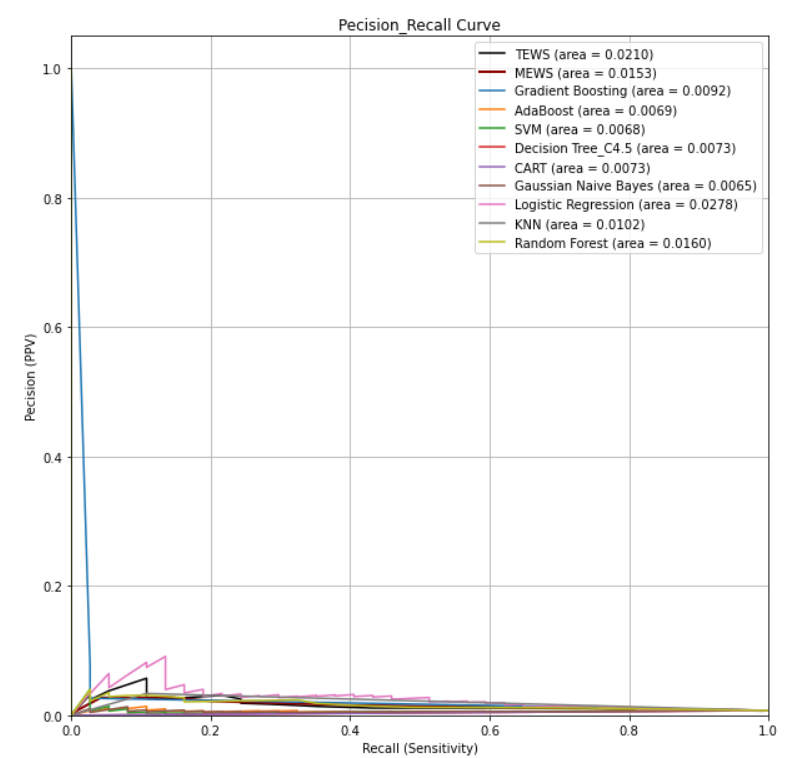


1. The PR curve of 6 Time windows with 30 features
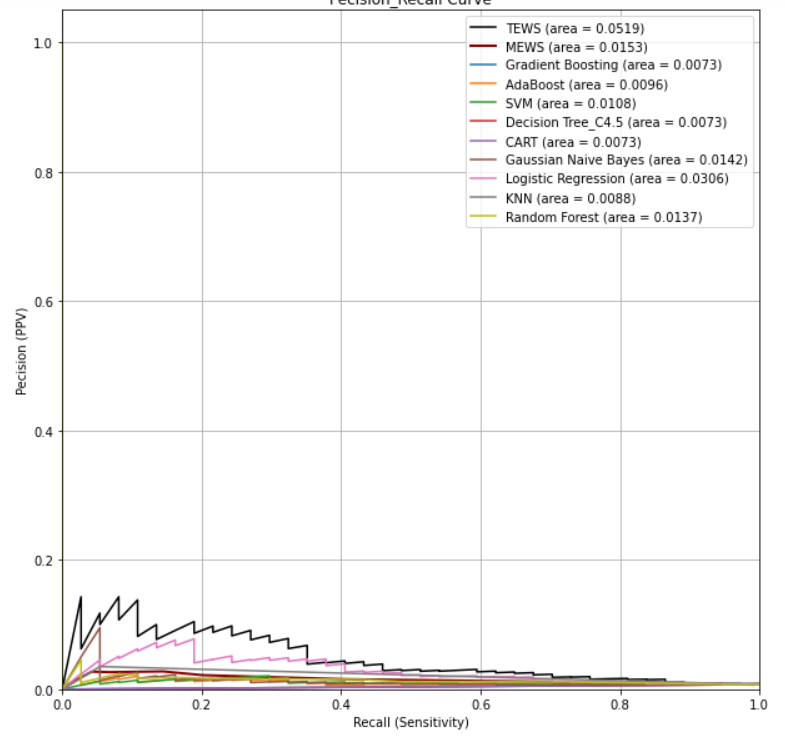

2. The PR curve of 6 Time windows with 5 selected features
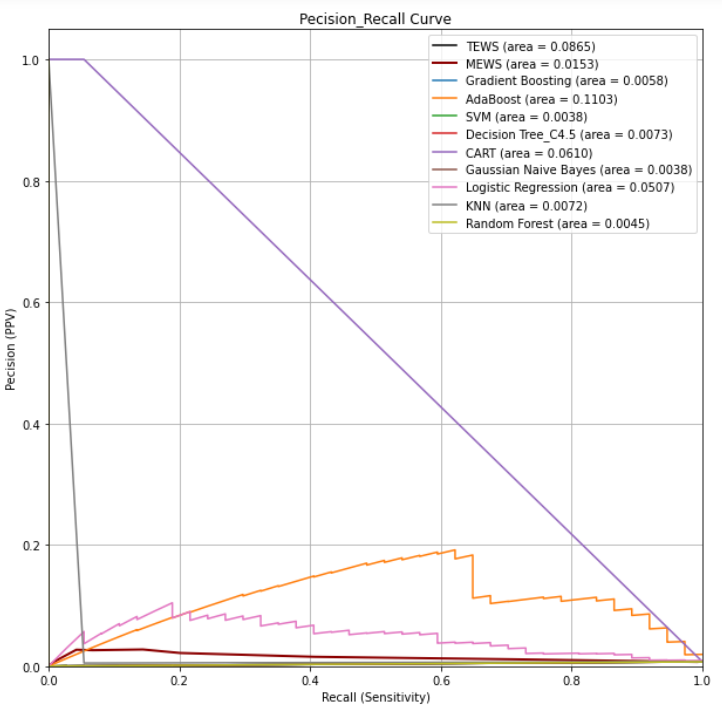

Supplement: Supplementary file 1 — Supplementary Information. [file 41598_2022_16195_MOESM1_ESM.docx]
